# Abusive spinal injury: imaging and updates

**DOI:** 10.1007/s00247-024-06043-y

**Published:** 2024-09-05

**Authors:** Betul E. Derinkuyu, Marguerite M. Caré, Kathi L. Makoroff, J. John Choi

**Affiliations:** 1 Department of Radiology and Medical Imaging, Cincinnati Children’s Hospital Medical Center, 3333 Burnet Avenue, Cincinnati, OH 45229 USA; 2 Mayerson Center for Safe and Health Children, Cincinnati Children’s Hospital Medical Center, Cincinnati, OH USA; 3 Department of Pediatrics, University of Cincinnati College of Medicine, Cincinnati, OH USA

**Keywords:** Abusive head trauma, Abusive spinal injury, Child physical abuse, Fracture, Ligamentous injury, Magnetic resonance imaging, Radiology, Spine

## Abstract

Child physical abuse has significant morbidity and mortality in the pediatric population. There is growing evidence that abusive spinal injury has been under-recognized, changing historical perceptions that these injuries are relatively uncommon. Increased utilization of MRI has been pivotal in recognizing that most abusive spinal injuries involve the soft tissues and ligaments or manifest as intrathecal blood products, which are often undetectable by radiography or CT. Detecting spinal injury in the work-up of non-accidental trauma improves management for abused children and their siblings (defined as siblings or other household members). This review highlights key points in the imaging literature of abusive spinal injury, describes typical patterns of injury, and addresses appropriate imaging practice for work-up.

**Graphical Abstract:**

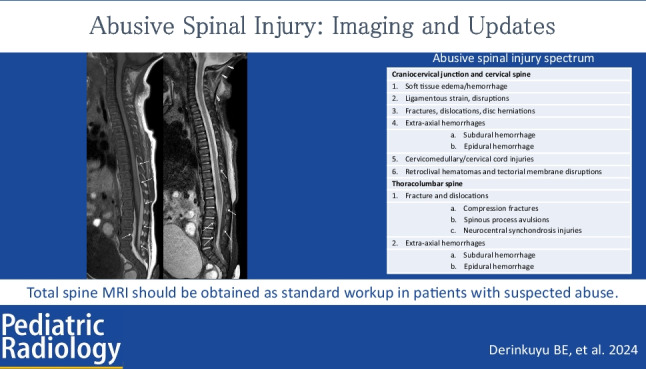

## Introduction

Child physical abuse is one of the leading causes of morbidity and mortality in the pediatric population. Literature regarding abusive spinal injury, once thought to be a relatively uncommon inflicted injury, has been expanding over the last two decades coinciding with increased utilization of cross-sectional imaging (e.g., magnetic resonance imaging [MRI]) in the work-up of inflicted craniospinal trauma. With improved understanding of the various mechanisms underlying inflicted craniospinal injuries, a recent consensus statement defining abusive head trauma (AHT) includes inflicted injuries to the craniocervical junction (CCJ) and spine under the scope of abusive head injuries [1]. Though epidemiological and demographic data are still coming to light, abusive spinal injury tends to occur in younger patients (less than 2 years of age) and results in longer hospital stays, incurring higher hospital costs versus children with accidental spinal injuries [2]. Although there is improved understanding of prevalence, mechanisms, and types of abusive spinal injury, diagnosis remains challenging due to various factors including misleading information from caregivers, inability of preverbal children to communicate, and a normal appearing initial skeletal survey. In addition, clinical assessment may be confounded due to concurrent AHT, which may mask or mimic spinal findings with potentially devastating consequences if undetected [3, 4].

Spinal injury in infants and young children can point towards abuse, especially when an adequate history is not provided to explain the injury [3]. As reported by Karmazyn et al., most abusive spinal injuries manifest as soft tissue and ligamentous injuries (10.1%) as well as spinal subdural (23.0%) and/or epidural (2.0%) hemorrhages rather than bony fractures (7.4%) which may be missed if the appropriate imaging examination is not performed [5]. Increased use of spine MRI has identified many types of inflicted spinal injuries and expanded our understanding of injury patterns; however, there remains debate and heterogeneity with imaging practice [4, 6, 7]. The recommendation of total spine MR imaging in cases of AHT has been made in several recent guidelines due to emerging evidence linking abusive spinal injury with AHT and cytotoxic brain edema/hypoxic ischemic injury (HII) [1, 6, 7]. Recognizing abusive spinal injury is crucial, as physical signs of spinal injuries are not always present on clinical exam, as they may be masked by coexistent intracranial injuries. Therefore, imaging findings may be the only identifiable abnormality [8]. Detailed discussion and awareness of these injuries are essential, not only for correct diagnosis, but also to guide management in suspected cases and to potentially strengthen medicolegal arguments. Most importantly, identifying any abusive injury can potentially save a child or sibling from further harm.

In this review, our objectives are three-fold. First, we will highlight key points in recent imaging literature of abusive spinal injury. Second, we will review typical abusive injury patterns of the developing spine. Third, we address the appropriate imaging work-up in cases of abusive spinal injury, considering the most common imaging findings based on recent literature review and heterogeneous resources across healthcare institutions.

## Imaging literature of abusive spinal injury

Historically, the first documented cases of abusive spinal injury date back to the 1950s [9, 10]. Since then, there has been increasing recognition of spinal injury in cases of inflicted injury, although prevalence remains difficult to determine. Based on the skeletal survey literature, spine fractures are rarely detected, with reported ranges from 0 to 3% [11–13]. In a meta-analysis reviewing 365 skeletal surveys of children younger than 2 years with suspected abuse, Kleinman et al. found that of 62% positive skeletal surveys, only ten exams had spinal fractures [14]. Similarly, in another series of skeletal surveys of 31 fatally abused infants, only 1 spinal fracture was detected out of a total of 165 skeletal fractures (51% ribs, 44% long bones, 4% hands/feet, 1% clavicles) [15]. The types of reported spinal fractures related to abusive spinal injury can vary widely, from subtle spinal compression deformities to fracture-dislocations with severe neurological injury from spinal cord compression or contusion [16].

With the increased utilization of cross-sectional imaging, other injury patterns have come to light. In a comprehensive literature review of spinal trauma spanning 1950 to 2009, Kemp et al. summarized findings in 25 children with abusive spinal injury from 19 case reports and case series [17]. The review consisted of 47 imaging studies (24 skeletal surveys, 7 spine computed tomography scans [CTs], and 16 spine MRIs). Among those cases, 48% of children had cervical spine injury (median age of 5 months), 48% of children had thoracolumbar spine injury (median age 13.5 months), and only one child had cervical and thoracic spine, as well as sacral injuries [17]. Based on their review, the authors recommended that all skeletal surveys should include lateral views of the spine to improve detection of fractures. They also recommended that any clinical or radiological suspicion of spinal injury warrants a spine MRI, especially for children undergoing a brain MRI for AHT [17].

Recent studies have focused on defining the prevalence of cervical spinal ligamentous injuries in cases of AHT and its associations [4, 6]. In a study by Kadom et al. of 74 children with head trauma (38 with AHT, 26 with accidental head trauma, and 10 with undefined head trauma) evaluated by brain and cervical spine MRI, 36.5% of cases had cervical injuries (mostly ligamentous), usually in association with intracranial abnormalities [6]. They reported a statistically significant causal relationship between cervical injuries, AHT and HII, and recommended cervical spine MRI evaluation with detection of any intracranial injury (e.g., hemorrhage, HII), if abuse were suspected [6]. After comparing cervical spine MRIs obtained in 67 children with AHT to 46 children with accidental trauma, all less than 48 months of age, Choudhary et al. found that cervical ligamentous injury was more common in children with AHT than with accidental trauma (78% vs. 46%) [4]. Spinal subdural hemorrhage (SDH) was also more frequent in the AHT cohort compared to the accidental trauma cohort (48% vs. 2%, respectively) [4].

More recently, Rabbit et al. reported an incidence of spinal injury of 59% on MRI in 76 children less than 5 years of age who were evaluated for AHT [7]. Among the 45 positive cases, the most common injury patterns were ligamentous injury (42%), posterior paraspinous muscular edema (38%), prevertebral soft tissue swelling (32%), spinal SDH (16%), spinal epidural hemorrhage (8%), bony injury (5%), cord edema (1%), and vertebral artery dissection (1%) [7]. Notably, they reported a statistically significant association between the presence of spinal SDH and retinal hemorrhages, noncontact head injuries, and a diagnosis of AHT, suggesting that the specific finding of a spinal SDH anywhere in the thecal sac supports a mechanism of severe acceleration and deceleration injury [7]. In a prospective study of 52 children less than 36 months of age with inflicted trauma, Baerg et al. reported a cervical spine injury incidence of 15.1% and reported statistically significant associations with retinal hemorrhages, shaking injury mechanism, lower GCS, brain infarcts, and HII. They hypothesized that stretching of the neuroaxis (e.g., brainstem, CCJ, cervical nerve roots) causes respiratory insufficiency and HII [18]. Another study by Jacob et al. of 89 children less than 5 years of age with AHT reported an incidence of spinal injury up to 69% with interspinous ligamentous injury seen most frequently [19]. The authors also reported a statistically significant relationship between cervical spine injury and HII of the brain noting that children with restricted diffusion on brain MR imaging were 6.22 (point estimate) times more likely to have cervical spine ligamentous injury on MR imaging [19].

## Anatomy, injury mechanism, and injury spectrum

### Craniocervical junction and cervical spine

Developmental and biomechanical differences between young children and adults result in a discrepant pattern of spinal injury following trauma [20–23]. For infants and young children, the cervical spine and CCJ are most at risk for accidental and inflicted injury due to various factors including a disproportionately large head to body volume ratio, poorly developed cervical and neck musculature to support ligamentous stabilizers of the CCJ, cartilaginous growth plates, and the osseocartilaginous morphology of the developing spine. Head to total body length ratio starts at approximately 1:4 at birth and progressively reduces to 1:7 in the adult [24]. Barriers to resist flexion-rotation forces, such as the upper cervical facet joints and uncovertebral joints, are shallow and underdeveloped contributing to increased spinal mobility and vulnerability to injury [25]. While these unique anatomical features of the developing cervical spine offer hypermobility and some degree of protection for the osseous spinal column, they put the underlying CCJ and cervical cord at risk for injury with excessive or repetitive stress. This may explain the relatively low incidence of spinal fractures observed in young children with both accidental and inflicted cervical spine trauma.

The consequences of any form of CCJ or cervical abusive spinal injury are varied, ranging from minor swelling to potentially devastating injuries. Failure to recognize unstable cervical ligamentous or osseous injuries could lead to repetitive spinal cord injury, chronic myelopathy, and/or kyphotic deformity with significant disability and/or mortality. Clinical outcomes are often worse in the context of multisystem trauma with significant morbidity contributed by concomitant brain injuries [19, 26, 27].

The imaging features of abusive spinal injury to the CCJ and cervical spine encompass (1) soft tissue edema and hemorrhage; (2) ligamentous strains and tears; (3) fractures, dislocations and disc herniations; (4) subdural and epidural hematomas; (5) injuries to the cervicomedullary junction and cervical cord; and (6) retroclival hematomas and disruptions of the tectorial membrane (Table 1).

**Table 1:** Abusive spinal injury spectrum

**Craniocervical junction and cervical spine**
1. Soft tissue edema/hemorrhage
2. Ligamentous strain, disruptions
3. Fractures, dislocations, disc herniations
4. Extra-axial hemorrhages
a. Subdural hemorrhage
b. Epidural hemorrhage
5. Cervicomedullary/cervical cord injuries
6. Retroclival hematomas and tectorial membrane disruptions
**Thoracolumbar spine**
1. Fracture and dislocations
a. Compression fractures
b. Spinous process avulsions
c. Neurocentral synchondrosis injuries
2. Extra-axial hemorrhages
a. Subdural hemorrhage
b. Epidural hemorrhage

#### Soft tissue injuries

**Fig. 1:**
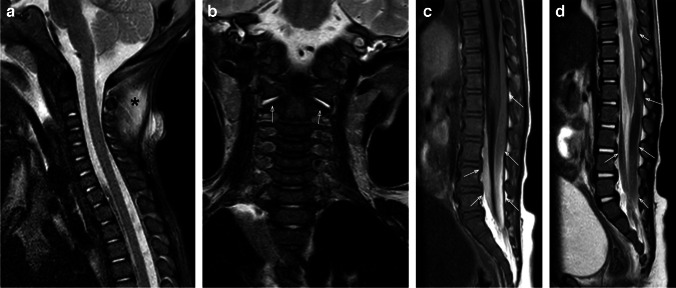
Fourteen-month-old boy presenting with lethargy and bruising. Sagittal STIR MR image (**a**) of the cervical spine demonstrates the nuchal ligament edema (*asterisk*) and dorsal soft tissue edema. Coronal T2-weighted MR image (**b**) shows fluid in both atlanto-axial joints (*arrows*). Sagittal T1-weighted (**c**) and T2-weighted (**d**) MR images of the lower spine show the T1-hyperintense and T2-hypointense subdural hemorrhage (*arrows*) extending inferiorly to the distal thecal sac

**Fig. 2:**
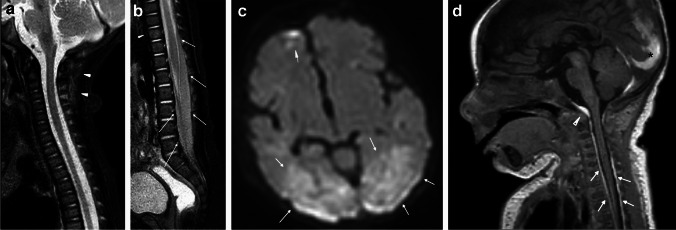
Two-month-old boy presenting with lethargy and possible seizure. Sagittal STIR MR image (**a**) of the upper spine demonstrates ligamentous edema in the nuchal ligament (*arrowheads*) and multiple interspinous ligaments. Sagittal STIR MR image (**b**) of the lower spine reveals the intermediate signal spinal SDH along the distal thecal sac (*arrows*). There is subtle marrow edema in the T10 vertebral body (*arrowhead*) consistent with contusion-type injury without loss of vertebral body height. Axial diffusion-weighted image (**c**) from a brain MRI demonstrates multifocal areas of diffusion restriction (*arrows*) compatible with areas of cytotoxic edema suggesting HII. A sagittal T1-weighted MR image (**d**) through the cervical spine obtained 1 week after presentation for ongoing seizures demonstrates redistribution of SDH with increased volume of T1-hyperintense subdural hemorrhage along the posterior falx (*asterisk*), in the posterior fossa, in the retroclival space (*arrowhead*), and within the upper spinal canal (*arrows*)

Posterior paravertebral soft tissue edema and ligamentous injury are the most commonly reported imaging findings of abusive spinal injury of the CCJ and cervical spine (Figs. 1 and 2), often coexisting with intracranial hemorrhage and HII (Fig. 2) [3, 6, 7]. Unfortunately, radiographic measurements used in adults to predict ligamentous instability have limited use in children; thus, MRI plays a crucial role in identifying soft tissue and ligamentous injury, especially when intracranial injury may mask an underlying spinal injury clinically, or the patient is immobilized and sedated limiting physical assessment and optimal radiographic views [28, 29].

**Fig. 3:**
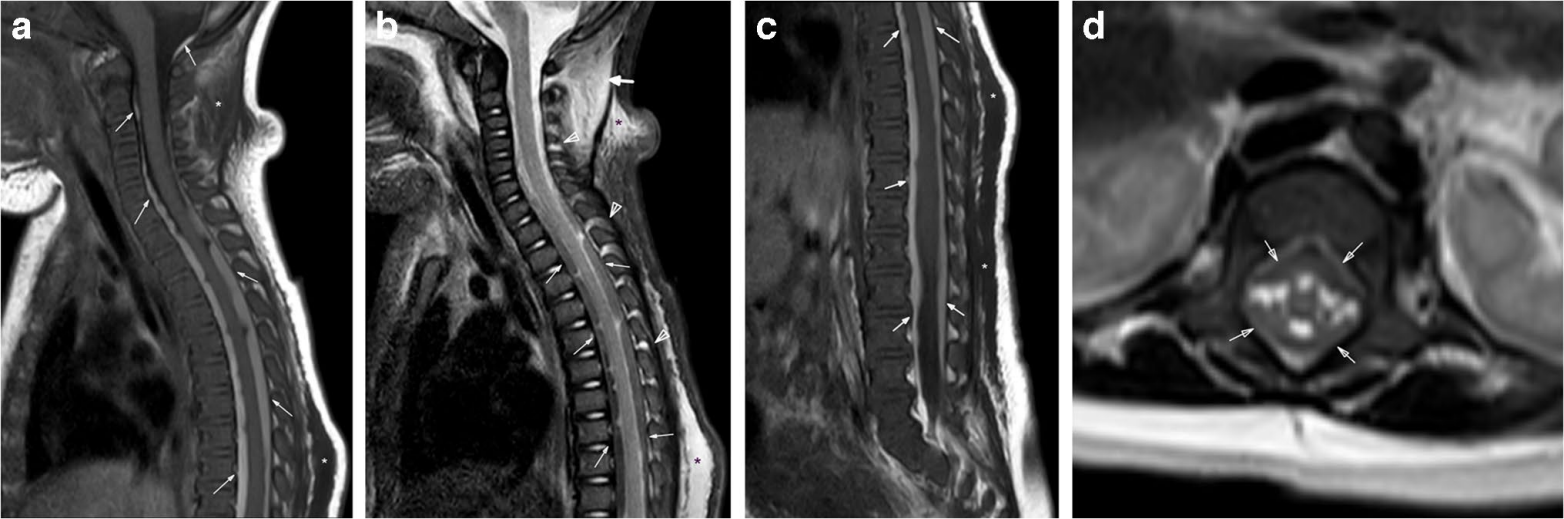
Four-month-old girl presenting with vomiting and seizure. Sagittal T1-weighted MR image (**a**) of the upper spine shows the hyperintense subdural hemorrhage (*arrows*) extending below along the spinal canal and nuchal ligament edema (*asterisk*). Sagittal STIR MR image (**b**) of the upper spine better demonstrates the dorsal soft tissue (*asterisk*) and ligamentous edema in the nuchal ligament (*thick arrow*), as well as interspinous ligamentous edema/injury (*arrowheads*). Note the STIR hypointense spinal SDH extending inferiorly from the level of CCJ with multiple loculations (*thin arrows*). Sagittal T1-weighted MR image (**c**) of the lower spine shows hyperintense subdural hemorrhage along the spinal canal (*arrows*). Note posterior subcutaneous soft tissue edema (*asterisk*). Axial T2-weighted MR image (**d**) from L1 level reveals the T2-hypointense subdural hemorrhage located at the anterior and posterior aspect of the spinal canal (*white arrows*)

**Fig. 4:**
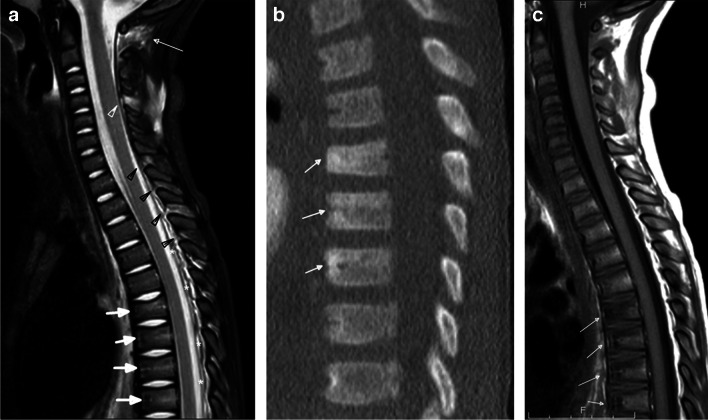
Two-year-old boy presenting with difficulty walking and unexplained bruising. Sagittal STIR MR image (**a**) of the upper spine demonstrates atlanto-axial ligamentous injury (*thin white arrow*) and interspinous ligamentous edema, widening of the interspinous distance, and disruption of the ligament flavum at C4-C5 (*white arrowhead*). There are also multilevel upper thoracic interspinous ligament edema with ligamentum flavum disruption (*black arrowheads*) and epidural fluid in the dorsal aspect of the mid thoracic spine (*white asterisks*). Note biconcave appearance of thoracic vertebral bodies with mild edema signal suggesting subtle compression fractures (*thick arrows*). Sagittal bone algorithm CT image (**b**) of the thoracic spine demonstrates loss of vertebral body height and sclerosis anteriorly of the T6, T7, and T8 vertebral bodies (*arrows*) with anterior wedging and T1-hypointense signal on the sagittal T1-weighted MR image (**c**) of the spine (*arrows*)

Most abusive ligamentous injuries of the infant involve the posterior ligamentous complex (Figs. 3 and 4), as a stronger network of anterior ligaments, including the transverse and alar ligaments and transverse band of the cruciform ligament provide anterior stability [3]. Injuries to the posterior ligamentous complex can manifest as edema, T2 prolongation, surrounding fat stranding and/or directly visible injury of the larger nuchal ligament or smaller atlanto-occipital membrane, posterior atlanto-axial ligament, or interspinous ligaments (Figs. 2, 3, and 4). Distraction-type injury to the capsular ligaments is suspected with joint effusion and increased distance of the occiput-C1 and C1-C2 joint spaces (Fig. 1). Anterior ligamentous complex injuries are less common with abusive spinal injury but can occur with severe inflicted trauma detectable by MRI as edema and or frank disruption of the apical and alar ligaments of the dens and/or cruciform ligament [3].

#### Osseous injuries

While bony injuries of the cervical spine are well-described in accidental trauma, they are less frequently reported in cases of abuse. If present in cases of abusive injury, cervical spine fractures are more likely to be associated with neurologic deficits compared to thoracolumbar vertebral body compression injuries. Cord injury can occur from retropulsed fracture or disc fragments, perched facets with severe hyperflexion injuries, or any fracture-dislocation resulting in severe cervical canal narrowing [30]. Spine MRI is important in these cases to evaluate the coexisting spinal cord injury.

#### Extra-axial hemorrhages

Though more commonly detected in the thoracolumbar spine, subdural and epidural hemorrhages can occur in the CCJ and cervical spine as a sequela of abuse. Epidural hemorrhages (EDH) are often the result of a localized bony or ligamentous injury. SDH are thought to reflect inferior run-off of blood products from the posterior fossa and are commonly seen in association with AHT with reported prevalence of 44–63% [31, 32]. Retroclival hematomas can be either epidural or subdural in location depending on depth relative to the tectorial membrane. Retroclival epidural hemorrhage represents blood products deep to the tectorial membrane and may imply CCJ injury with potential instability, especially if there is an associated tectorial membrane disruption. On the other hand, retroclival subdural collections are located superficial to the tectorial membrane and deep to the arachnoid membrane, and more likely represents run-off of intracranial blood products (Figs. 2, 3, and 4) [33]. The myodural bridge complex is theorized to represent the anatomical link between AHT, CCJ injury, and spinal SDH [4, 34]. Myodural bridges are a complex network of connective tissues extending from the suboccipital musculature to the cervical spinal dura mater, which functions to transmit tensile forces of the musculature during neck flexion and extension contributing to CSF flow and maintaining patency of the spinal canal at the CCJ [35]. In a severe flexion injury, acute traction on the myodural bridge complex is theorized to cause a rent in the dura-arachnoid interface forcing open the potential subdural space of the CCJ and creating a path for caudal migration of intracranial subdural blood products [4, 34, 36–38].

#### Cervicomedullary and cervical cord injuries

**Fig. 5:**
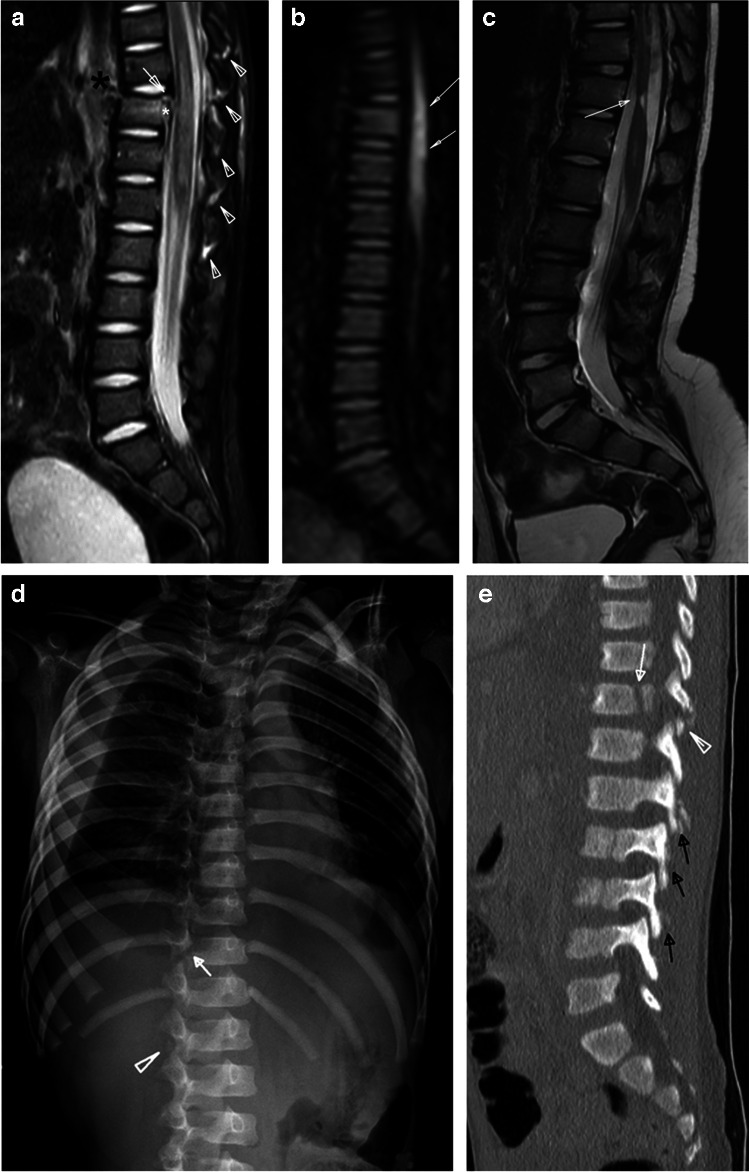
Two-year-old boy with multiple bruises and not moving lower extremities. Sagittal STIR MR image (**a**) of the lower spine demonstrates fracture of the posterior-superior vertebral body of T11 with disruption of the posterior longitudinal ligament (*arrow*). There is a small volume epidural hemorrhage dorsal to the fracture (*white asterisk*) and large volume retroperitoneal hemorrhage (*black asterisk*) overlying a disrupted anterior longitudinal ligament. Multilevel interspinous ligamentous edema and widening is noted at the thoracolumbar junction (*white arrowheads*). Acute injury to the spinal cord is visible on the sagittal STIR MR image (**a**) spanning T10-T12 as T2 prolongation and confirmed on a sagittal diffusion image (**b**) of the cord (*arrows*). A 6-month follow-up sagittal T2-weighted MR image (**c**) of the cord demonstrates sequelae of the cord injury with myelomalacia (*arrow*) at site of the previous injury. Oblique radiograph of the ribs (**d**) demonstrates subtle lucency through the right T11 neurocentral synchondrosis (*arrow*) and heterotopic bone formation adjacent to the right L1 facet joint (*arrowhead*), which were overlooked on the initial skeletal survey, but correspond with the interspinous edema and widening seen on prior MR (**a**) and fractures better seen by CT. Sagittal bone algorithm reconstruction from an abdominal CT with contrast (**e**) demonstrates the neurocentral synchondrosis fracture (*arrow*), facet injury (*arrowhead*), and heterotopic bone formation at multiple levels of the lumbar spine (*black arrows*)

Brainstem and upper cord injuries may be incurred from repetitive flexion–extension, traction, or cord ischemia, and may cause respiratory failure and HII. Reported prevalence of cervical cord injuries in inflicted spine injury ranges from <1% to 10% [39]. Historically, clinically diagnosed cord injuries without evidence of fracture or malalignment on radiography or CT were referred to as spinal cord injury without radiographic abnormality (SCIWORA), an outdated term in the era of MRI where acute and chronic cord injuries can be visualized directly (Fig. 5) [40]. Increased utilization of MRI will likely improve our understanding of mechanisms underlying cord injury through detection of ligamentous and subtle osseous anomalies that would have been missed by radiography or CT.

### Thoracolumbar spine

Anatomically, there are a few considerations for the developing thoracolumbar spine which result in unique patterns of injury compared to adults. The developing vertebral bodies have a vertebral body nucleus surrounded by cartilaginous end plates, similar in morphology to growth plates elsewhere in the skeleton. The ossified vertebral body is separated from the pedicles by neurocentral synchondroses, potential sites of injury which may result in antero- or retro-pulsion of the vertebral body nucleus, dependent on mechanism of injury. The immature spinous processes are capped by a growth plate, making them prone to osteocartilaginous- and avulsion-type injuries. Reported mechanisms of inflicted thoracolumbar spinal injury include repetitive hyperflexion, hyperextension, and axial loading, often associated with rotational forces. These mechanisms can result in varying patterns of injury to the growth plates depending on the direction and magnitude of the applied forces and patient age.

Imaging findings of abusive spinal injury of the thoracolumbar spine include (1) fractures, dislocations, and, uncommonly, disc herniations as well as (2) extra-axial hemorrhages (Table 1).

#### Fractures and dislocations

The most commonly reported inflicted osseous thoracolumbar injuries are compression fractures of the vertebral bodies (Fig. 4). Other unique patterns of thoracolumbar spinal injury are described in abuse cases, especially in young children, such as neurocentral synchondrosis injuries (Fig. 5), facet disruption, spinous process avulsions, and fracture-dislocations. Each pattern has characteristic imaging features and may require different management approaches.

**Fig. 6:**
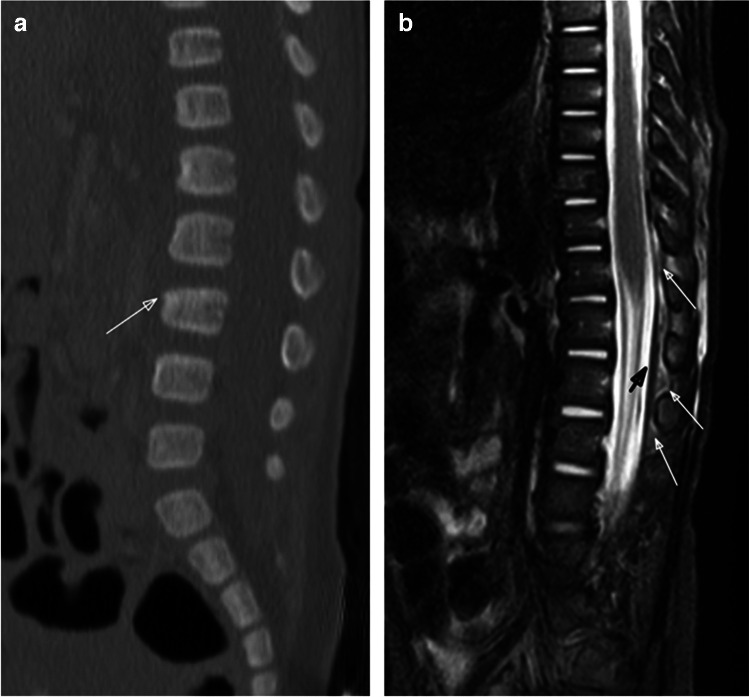
Five-month-old boy presents with bruising. Sagittal bone CT reconstruction of the lumbar spine from an abdominal CT (**a**) shows anterior wedging of the L3 vertebral body (*arrow*). Sagittal STIR MR image (**b**) demonstrates epidural fluid/hemorrhage (*white arrows*) and thin subdural hemorrhage (*black arrow*), as well as multilevel interspinous ligament injury/edema and subcutaneous soft tissue edema. No associated marrow edema corresponding to the L3 vertebral body wedging

Vertebral compression fractures can be detected with a skeletal survey and are more evident on lateral radiographs. However, radiographs underestimate the number of injuries and, thus, cross-sectional imaging modalities like CT and MRI play important roles in their diagnosis and evaluation (Figs. 4, 5, and 6). Fluid-sensitive MRI sequences (e.g., sagittal short tau inversion recovery (STIR)) are important to detect marrow edema when the loss of vertebral body height is subtle by skeletal survey or CT (Figs. 2 and 4). Paraspinal calcifications and heterotopic bone formation on skeletal survey or CT are important clues to help detect a healing spinal injury (Fig. 5). Levin et al. identified paravertebral calcification in six of seven patients with thoracolumbar fractures secondary to abusive trauma, two of whom were paraplegic from their injuries [41]. Most thoracolumbar fracture-dislocations occur around the thoracolumbar junction often with compression fracture deformity suggesting a hyperflexion-type mechanism of injury with great enough force to cause both bony, ligamentous and sometimes cord injury (e.g., transverse or ventral load on the low back or abdomen) (Figs. 4, 5, and 6). Therefore, it is important to examine the thoracolumbar junction carefully for heterotopic bone and/or widening of the neurocentral synchondroses on oblique views of the ribs, where they can be detected the most readily (Fig. 5).

#### Extra-axial hemorrhages

**Fig. 7:**
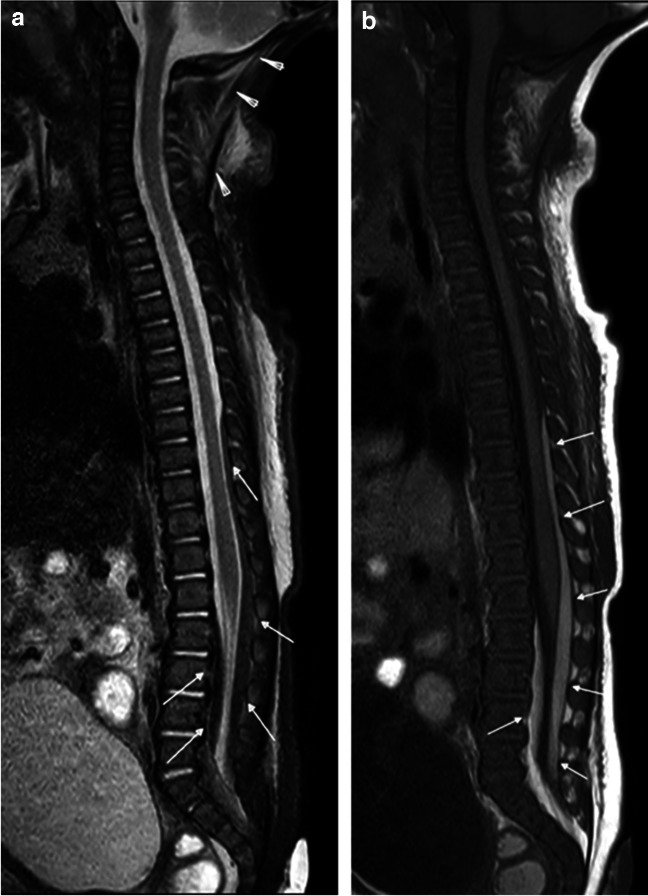
Three-month-old girl presenting with seizure, vomiting, and scalp swelling. Sagittal STIR (**a**) and T1-weighted (**b**) MR images of the total spine demonstrate posterior ligamentous complex injury with extensive edema in the nuchal ligament (*arrowheads*), as well the atlanto-occipital, atlanto-axial, and cervical interspinous ligaments. Extensive edema in the subcutaneous soft tissues of the suboccipital region and dorsal soft tissues overlying the thoracic spine. There is also thoracolumbar SDH (*white arrows*) which is T2-hypointense and T1-hyperintense. Additional work-up of this patient (not shown) revealed findings of AHT with mixed attenuation SDH and HII, calvarial fractures, multiple healing rib fractures, and a right femur metaphyseal corner fracture

As outlined previously, thoracolumbar spinal subdural collections of abuse are presumed to represent run-off from an intracranial SDH (Figs. 1, 2, 3, and 7). However, localized injury of a radicular artery or vein is also a potential source for SDH. In a review highlighting the various types of spinal injuries detected in cases of suspected AHT, Karmazyn et al. reported findings from 148 children who obtained total spine MRI for work-up of suspected AHT. In 47/148 (31.8%), there were major findings (defined as SDH, EDH, ligamentous injury, and spine fracture not identified by skeletal survey) of which 24/47 (51.1%) of the injuries were localized to the thoracolumbar spine, including 23 cases of SDH, two cases of EDH, and nine vertebral fractures, five of which were not identified by skeletal survey. They concluded that whole-spine MRI must be performed for patients with suspected AHT; otherwise, a substantial number of thoracolumbar injuries could be missed [5].

## Spinal imaging protocol for abuse

Skeletal survey remains the first-line imaging modality for radiologic assessment for suspected abuse [42]. Lateral views of the cervical, thoracic, and lumbosacral spine should be obtained as part of the standard imaging protocol [43]. Additional radiographs may be obtained as deemed necessary by a radiologist before the patient is released from the department, as is routine practice in the authors’ institution. It should be noted that evaluation of the CCJ can be particularly challenging by radiography due to overlapping structures, limited patient cooperation, and potentially fixed positioning from immobilization. If there is any clinical or radiographic suspicion for head or CCJ injury, one should have a low threshold for obtaining cross-sectional imaging. At the authors’ institution, CT head imaging obtained as part of a trauma protocol includes the upper cervical spine to the level of C2 to image the CCJ and evaluate for associated injury [44]. In addition, sagittal soft tissue reconstructions through the CCJ are valuable for detecting ligamentous injury, retroclival fluid, and prevertebral edema. Therefore, multiplanar reconstructions are invaluable for detecting injuries that may be very subtle on axial images.

### Heterogeneity of practice

Beyond the routinely performed skeletal survey and head CT in cases of suspected abuse, spine MRI utilization varies widely and is reported anywhere between 4.3–84.3% across the USA [45]. Depending on findings on the initial survey, physical examination, time of presentation, resource availability (e.g., sedation, MR coverage, child abuse physicians), patient age, and clinical stability, the next steps in imaging work-up may be met with various challenges intrinsic or extrinsic to a radiology department. Based on a 2022 survey of 597 members of the Ray E. Helfer Society, an international honorary society of physician leaders in child abuse, 107 respondents reported that the most common reason for not performing whole-spine MRI was lack of clear local or national guidelines on spine imaging in cases of AHT (67.7%) followed by the recommendation meeting resistance by another service (49.2%), potential need for sedation (46.2%), and long scanning times (42.5%) [46].

### Consensus imaging recommendations

In 2017, authors of the American College of Radiology (ACR) appropriateness criteria for suspected physical abuse in a child recommended that MRI of the cervical spine is “usually appropriate” in cases of suspected AHT and should be considered in concert with a planned MRI of the brain [42]. However, screening MRI of the total spine in suspected AHT was rated as “may be appropriate,” noting that whole-spine MRI resulted in increased detection of thoracolumbar SDH, although this rarely changed management, and rarely resulted in cord compression or long-term complications [42].

Multiple societies have more recently published their consensus statements for spinal imaging and have agreed that in cases highly suspicious for inflicted trauma, MR imaging of the entire spine should be obtained [1, 3, 7, 34, 47, 48]. Canty et al., on behalf of the Enhancing Neuroimaging Genetics through Meta-analysis (ENIGMA) Child Abuse Working Group, a collaborative network of researchers working together on large-scale studies integrating data from 70 institutions worldwide, advocated for whole-spine MRI in suspected AHT, noting that clinically significant and management altering spinal cord and spine injuries have likely been overlooked in the literature and would continue to be under-identified if whole-spine MRI is performed on a case-by-case basis or the ACR recommendations remain “may be appropriate” instead of changing the designation to “usually appropriate” [46, 49]. The main arguments agreed upon by various societies and working groups for whole-spine MRI include low number of spinal injuries detectable by skeletal survey and CT (< 10%) and a growing understanding that most of these injuries (ligamentous and run-off SDH) would only be detected by whole-spine MRI [3]. In addition, MRI may identify additional injuries inconsistent with provided histories, better characterize potential mechanisms of injury, and strengthen medicolegal and forensic abuse investigations [48].

### Summary of imaging recommendations

If possible, we also recommend brain and total spine MRI in suspected abuse cases, as recommended by the ENIGMA Child Abuse Working Group and ESPR child abuse taskforce [5, 46, 48]. While there are some arguments against spinal imaging, including considerations of cost and lack of surgical intervention needed in most cases, we liken identifying a finding of inflicted spinal abuse to that of detecting a spinal metastasis [50]. Its presence should be identified and flagged for the appropriate clinical teams to formulate the best comprehensive plan for patient care.

Considering practice and resource heterogeneity, a few generalizable imaging recommendations are suggested:

- A negative skeletal survey or CT of the spine does not rule out abusive spinal injury, and an MRI should be obtained based on clinical suspicion for suspected abuse [8].
- If a chest, abdomen, and/or pelvis CT is obtained as part of an abuse work-up, spine imaging should be reconstructed from the source images, without need for a repeat CT scan.
- When spine MRI imaging is obtained, it should ideally be performed within 72 h from presentation, before potential resorption of blood products or resolved soft tissue/ligamentous edema.

### Imaging protocols

Spine imaging protocols for abusive injury vary between institutions and numerous studies, and reports have listed relative strengths of various sequences. For example, Canty et al. recommended obtaining sagittal T1-weighted, sagittal STIR, axial T1-weighted, and axial T2-weighted sequences as a minimum whole-spine MRI protocol to be performed in cases of suspected abuse [46]. Our departmental protocol for a total spine MRI includes axial and sagittal T2-weighted images of the spine, large field of view (FOV) coronal T2 for evaluation of the soft tissues, sagittal T1-weighted images of the spine, sagittal STIR of the spine, and 3D-T1-weighted gradient echo (GRE) sequences of the lumbar spine for evaluation of the cortical bone [51]. In our experience, sagittal STIR and T1-weighted sequences of the whole spine with small FOV for the cervical region are most effective at identifying the commonly reported findings of inflicted injury (e.g., ligamentous and soft tissue edema; EDH and SDH; marrow edema and fractures) and should be prioritized (Fig. 7). A T2 Dixon technique could also be obtained in lieu of a STIR and may offer benefits for detection of marrow and ligamentous edema with typically more uniform fat suppression over STIR. Additional sequences should be obtained and added as problem-solving tools considerate of magnet and sedation times.

## Conclusion

Advances in imaging and widespread utilization of MRI have increased our knowledge and understanding of abusive spinal injury and its associations with other types of abuse. With the understanding that most of these injuries are ligamentous or hemorrhagic and often only detectable by MRI, we hope to add to the growing literature and consensus that total spine MRI should be obtained as standard work-up in patients with suspected abuse. Our hope is that improved detection and understanding will lead to more optimal management for these patients and their unfortunate injuries.

## Data Availability

The image set displayed in this study is not publicly available. However, de-identified data can be obtained upon request.
